# Fossilization of melanosomes via sulfurization

**DOI:** 10.1111/pala.12238

**Published:** 2016-04-01

**Authors:** Maria E. McNamara, Bart E. van Dongen, Nick P. Lockyer, Ian D. Bull, Patrick J. Orr

**Affiliations:** ^1^ School of Biological, Earth and Environmental SciencesUniversity College CorkNorth MallCorkIreland; ^2^ School of Earth, Atmospheric and Environmental SciencesWilliamson Research Centre for Molecular Environmental ScienceUniversity of ManchesterOxford RoadManchesterM13 9PLUK; ^3^ School of ChemistryManchester Institute of BiotechnologyUniversity of Manchester131 Princess StManchesterM13 9PLUK; ^4^ Organic Geochemistry UnitSchool of ChemistryUniversity of BristolBristolBS8 1TSUK; ^5^ UCD School of Earth SciencesUniversity College DublinBelfield Dublin 4Ireland

**Keywords:** melanin, melanosomes, sulfurization, fossil preservation, fossil colour, Libros biota

## Abstract

Fossil melanin granules (melanosomes) are an important resource for inferring the evolutionary history of colour and its functions in animals. The taphonomy of melanin and melanosomes, however, is incompletely understood. In particular, the chemical processes responsible for melanosome preservation have not been investigated. As a result, the origins of sulfur‐bearing compounds in fossil melanosomes are difficult to resolve. This has implications for interpretations of original colour in fossils based on potential sulfur‐rich phaeomelanosomes. Here we use pyrolysis gas chromatography mass spectrometry (Py‐GCMS), fourier transform infrared spectroscopy (FTIR) and time of flight secondary ion mass spectrometry (ToF‐SIMS) to assess the mode of preservation of fossil microstructures, confirmed as melanosomes based on the presence of melanin, preserved in frogs from the Late Miocene Libros biota (NE Spain). Our results reveal a high abundance of organosulfur compounds and non‐sulfurized fatty acid methyl esters in both the fossil tissues and host sediment; chemical signatures in the fossil tissues are inconsistent with preservation of phaeomelanin. Our results reflect preservation via the diagenetic incorporation of sulfur, i.e. sulfurization (natural vulcanization), and other polymerization processes. Organosulfur compounds and/or elevated concentrations of sulfur have been reported from melanosomes preserved in various invertebrate and vertebrate fossils and depositional settings, suggesting that preservation through sulfurization is likely to be widespread. Future studies of sulfur‐rich fossil melanosomes require that the geochemistry of the host sediment is tested for evidence of sulfurization in order to constrain interpretations of potential phaeomelanosomes and thus of original integumentary colour in fossils.

The study of fossil colour is an emerging field in palaeontology and evolutionary biology. Much research to date has focused on the preservation of morphological and chemical evidence of melanin in fossils and its implications for reconstructions of the original colouration of ancient animals (Clarke *et al*. 2008; Li *et al*. 1984, 1997; Zhang *et al*. 2010; Carney *et al*. 2012; Lindgren *et al*. 2011), the evolutionary history of visual signalling (Clarke *et al*. 2008; Li *et al*. 1984, 1997; Zhang *et al*. 2010; Carney *et al*. 2012) and of physiology (Li *et al*. 1997) and the fidelity of the fossil record (Glass *et al*. 2014, 2012; Lindgren *et al*. 2011). Micron‐sized granules of melanin, i.e. melanosomes, have been reported from various vertebrate and invertebrate fossils ranging in age from the Devonian to the Eocene, i.e. feathers (Vinther *et al*. 1989; Barden *et al*. 2011; Knight *et al*. 2011; Carney *et al*. 2012), birds (Clarke *et al*. 2008; Zhang *et al*. 2010; Wogelius *et al*. 2008), dinosaurs (Clarke *et al*. 2008; Li *et al*. 1984, 1997; Zhang *et al*. 2010), fish (Lindgren *et al*. 1998), marine reptiles (Lindgren *et al*. 2011), mammals (Li *et al*. 1997) and squid (Glass *et al*. 2014, 2012). These fossil microstructures are superficially similar to some bacteria in terms of their morphology and size (Vinther *et al*. 2010; Zhang *et al*. 2010; Knight *et al*. 2011) but can be most plausibly interpreted as fossil melanosomes based on details of their arrangement, location, precise context within soft tissues, and in particular their geochemistry (for further discussion see Barden *et al*. 2014; Moyer *et al*. 2010). Certain trace elements (e.g. Cu, Zn, Ca, and S) have been proposed as biomarkers for melanin (Wogelius *et al*. 2008; Manning *et al*. 2000b). Fossil melanosomes can also retain organic geochemical evidence for the melanin molecule itself (Glass *et al*. 2014, 2012; Lindgren *et al*. 1998, 2011). Somewhat surprisingly, despite such intensive study, current understanding of the taphonomy of melanin and melanosomes is limited. In particular, no mechanism has been proposed to explain the widespread preservation of melanin and melanosomes in fossils. Maturation experiments using feathers from extant birds (McNamara *et al*. 2013) and other taxa (Colleary *et al*. 2010) have shown that melanosome size is altered during diagenesis. Chemical analyses of melanosomes preserved in fossil fish have suggested that melanin is preferentially preserved while other original chemical components of the organelles, such as proteins and lipids, are almost completely degraded (Lindgren *et al*. 1998). Analyses of melanosomes preserved in Jurassic squid indicate diagenetic cross‐linking (Glass *et al*. 2014). Previous studies have referred to the high recalcitrance of melanin (Glass *et al*. 2014): it is a highly cross‐linked inert polymer of dihydroxyindole and dihydroxyindole carboxylic acid (McGraw 2010) that resists microbial degradation (Goldstein *et al*. 2013), oxidation and hydrolysis by acids and bases (Riley 2013b). It is unclear, however, whether such intrinsic chemical properties of the melanin molecule alone are responsible for widespread preservation of melanin and melanosomes in fossils, or whether other geochemical processes contribute to preservation.

There is increasing evidence that the organic preservation of many non‐biomineralized tissues, such as the cuticle of fossil arthropods and plants and the periderm of graptolites, reflects *in situ* polymerization of authochthonous lipids during diagenesis (Briggs 1999; Stankiewicz *et al*. 2012; Gupta *et al*. 1996, 2011; Manning *et al*. 2000a, 2002; Dutta *et al*. 2006; Gupta & Briggs 1998; Barden *et al*. 2011; Edwards *et al*. 2011). This process generates an aliphatic component similar to Type I and II kerogens (Briggs 1999) that can be identified in fossils, typically as a series of alkane/alkene doublets. Indeed, such aliphatic compounds are present in melanosome‐bearing soft tissues from various fossils (Barden *et al*. 2011, 2014; Glass *et al*. 2012), suggesting that polymerization processes are also involved in the preservation of melanin and melanosomes. *In situ* polymerization of organic molecules may be promoted via the incorporation of S (in the form of sulfides or polysulfides) from the surrounding environment (de Graaf *et al*. 2015; Schouten *et al*. 2014; Sinninghe Damsté *et al*. 1992, *b*, 2004; Kok *et al*. 2003, *b*; Werne *et al*. 1984; van Dongen *et al*. 1998a, *b*). This process creates resistant macromolecules comprising carbon chains cross‐linked by (poly‐)sulfide bridges and can be completed during very early diagenesis (i.e. within 10 ka; Kok *et al*. 2003; Werne *et al*. 1984; Sinninghe Damsté *et al*. 2004). Further, *in situ* polymerization via sulfurization, also known as natural vulcanization (Tegelaar *et al*. 1994), can enhance the preservation potential of organic materials on geological timescales (Sinninghe Damsté *et al*. 1992; Melendez *et al*. 2006, *b*). It has been invoked to explain the preservation of very labile organic components, such as carbohydrates (van Kaam‐Peters *et al*. 2000; Sinninghe Damsté *et al*. 2006; Kok *et al*. 2003; van Dongen *et al*. 1998a, *b*, 1999), as well as soft tissues in fossils as sulfur‐rich organic remains (McNamara *et al*. 2012, 2014). Intriguingly, many examples of melanosome‐bearing soft tissues in fossils contain organic sulfur compounds (OSCs; Glass *et al*. 2014, 2012; Lindgren *et al*. 2011) or are associated with elevated levels of sulfur (Barden *et al*. 2011, 2014; Lindgren *et al*. 1998; Pinheiro *et al*. 2013) , although the latter does not preclude preservation of certain S‐bearing amino acids such as cysteine (Barden *et al*. 2011, 2014) or of S‐bearing phaeomelanins (rather than eumelanin, which does not contain S) (Lindgren *et al*. 2011). It is therefore possible that preservation of melanosomes in many fossils is promoted by diagenetic sulfurization. Indeed, the possibility of diagenetic incorporation of sulfur into melanosomes has been posited (Lindgren *et al*. 2011), although this has been questioned (Colleary *et al*. 2010). The latter study of diverse fossil taxa reported clustering of time of flight secondary ion mass spectrometry (ToF‐SIMS) chemical data on the basis of taxonomy, rather than depositional setting; these data were interpreted as evidence that diagenetic sulfurization of eumelanin is ‘negligible’. However, data derived from ToF‐SIMS analysis cannot differentiate unequivocally between preserved phaeomelanin and diagenetically altered eumelanin (Colleary *et al*. 2010). This uncertainty regarding the nature of S‐bearing moieties in fossil melanosomes limits our understanding of melanosome taphonomy and, critically, our ability to identify fossil phaeomelanin, and thus has important implications for inferences of original colour in fossils. Complementary geochemical analyses of both fossil melanosomes and associated sediments are therefore required in addition to ToF‐SIMS analyses to determine the origins of S‐bearing moieties in fossil melanosomes.

We investigated sulfur‐rich, organically preserved melanosome‐like microstructures preserved in fossil frogs, and the host sediment, from the Late Miocene Lagerstätte of Libros, NE Spain (Fig. 1A). The soft tissues of the frogs include dark brown to black layers of carbonaceous, spherical to ovoid microstructures previously interpreted as fossil bacteria (Fig. 1A–B; McNamara *et al*. 2012, 2012). The high concentration of sulfur (*c*. 10% S) in the fossil microstructures has been attributed to sulfurization, but this has not yet been confirmed using organic geochemical techniques (McNamara *et al*. 2012). Geochemical data have provided critical evidence that similar microstructures associated with the soft tissue outlines of other vertebrate fossils represent preserved melanosomes and not fossil bacteria (Glass *et al*. 2014, 2012; Lindgren *et al*. 1998, 2011). In light of this, we reassessed our initial interpretations of the nature of the Libros microstructures and tested previous hypotheses that sulfurization contributed to their preservation. Here, we couple detailed analysis of the morphology and spatial context of the microstructures with new geochemical data from pyrolysis gas chromatography mass spectrometry (Py‐GCMS), fourier transform infrared spectroscopy (FTIR) and ToF‐SIMS. Our new results provide strong evidence that the Libros microstructures are not fossil bacteria but melanosomes and that sulfurization played an important role in their preservation.

**Figure 1:**
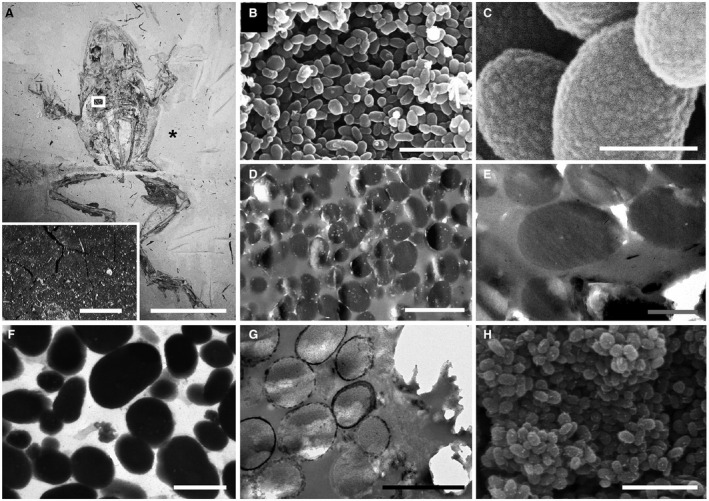
Melanosome‐like microbodies in the Libros frogs. A, MNCN 63663; inset, detail of thin, dark brown carbonaceous film defining soft tissues in area indicated; asterisk indicates region of sediment analysed in Fig. 4E. B–H, scanning (B–C, H) and transmission (D–G) electron micrographs showing details of melanosome‐like microbodies in the brown layer. B–C, densely packed melanosome‐like microbodies (B) with detail of surface texture (C). D–F, unstained (D–E) and stained (F) TEM sections of microbodies showing uniform electron density; note internal vacuoles in microbodies in D. G–H, unstained sections of microbodies immediately adjacent to the phosphatized skin showing electron‐dense margin of calcium phosphate (G) and nanocrystalline surface texture (H). Scale bars represent: 50 mm (A); 1 mm (inset in A); 5 μm (B, F); 500 nm C, E–F); 1 μm (D); 2 μm (G).

## Geological background

The Libros frogs are hosted within organic‐rich laminated mudstones (oil shales) of the Libros Gypsum Unit (Vallesian) which outcrops *c*. 25 km SE of Teruel in NE Spain. The unit was deposited in deep‐water zones of a stratified freshwater lake (Anadón *et al*. 1992); low oxygen isotope values from primary gypsum deposits and abundant gammacerane in the laminated mudstones indicate persistent high sulfide levels and intense bacterial sulfate reduction in the monimolimnion (de las Heras *et al*. 2003a; Ortí *et al*. 2012). The kerogen from the laminated mudstones contains abundant OSCs indicative of extensive sulfurization (del Río *et al*. 2003b) and there is no evidence for the presence of benthic microbial mats (McNamara *et al*. 2009).

## Material and method

### Fossil specimens

Most specimens (n = 73) are preserved as well‐articulated skeletons with soft tissues defined by a combination of pale‐toned phosphatized skin and dark‐toned layers (one thick layer internal to the phosphatized skin, and one thin layer, external) of densely packed carbonaceous microbodies *c*. 0.4–1.0 μm wide (Fig. 1A; McNamara *et al*. 2012, 2012). The context, morphology and ultrastructure of the fossil microbodies have been described previously (McNamara *et al*. 2012). In brief, the microbodies are restricted to the outline of the soft tissues and most are located internal to the phosphatized skin where they envelop the bones and stomach contents (McNamara *et al*. 2012). The microbodies typically exhibit granular to botryoidal surface nanotextures (Fig. 1B–C) and are usually uniformly electron‐dense in unstained (Fig. 1D–E) and stained (Fig. 1F) TEM sections; electron‐lucent inclusions may be apparent (Fig. 1D–E). Microbodies located immediately adjacent to the phosphatized skin (i.e. within *c*. 5 μm) exhibit a more electron‐dense margin interpreted as a diagenetic precipitate of calcium phosphate (Fig. 1G), the phosphate ions for which were derived from the decaying skin (which is rich in calcium phosphate *in vivo*). These microbodies exhibit a nanocrystalline texture on their surfaces (Fig. 1H) and are rich in Ca and P in addition to C.

##### Institutional abbreviation

MNCN, Museo Nacional de Ciencias Naturales, Madrid.

### Electron microscopy

Small (1–2 mm^2^) samples of the dark layers were dissected using sterile tools and prepared for scanning‐ and transmission‐electron microscopy (SEM and TEM) as in McNamara *et al*. (2012). Samples for SEM were sputter coated with gold and examined using a Hitachi S‐3500N variable pressure SEM at an accelerating voltage of 15 kV. For TEM, unstained and stained ultrathin (80–90 nm thick) microtome sections were placed on Cu grids and examined using a JEOL 2000TEMSCAN operating at 80 kV and using an objective aperture of 10 μm diameter.

### Py‐GCMS

Samples of the fossil tissues and host sediment were collected and stored in aluminium foil and crushed in a mortar. A 10 μL measure of tetramethylammonium hydroxide (TMAH) solution was added to each sample to allow thermochemolysis. Py‐GCMS was performed using a Chemical Data Systems (CDS) 5200 series pyroprobe pyrolysis unit attached to an Agilent 7890A gas chromatograph (GC) fitted with an HP‐5MS fused column (J&W Scientific 5% diphenyl‐dimethylpolysiloxane; 30 m, 0.25 mm i.d.; 0.25 μm film thickness) and an Agilent 5975C MSD single quadrupole mass spectrometer operated in electron ionization (EI) mode (EI source temperature 230°C, MS quadrupole 150°C and interface 280°C) with helium as a carrier gas (1 mL/min). Samples were pyrolysed in a quartz tube at 600°C for 20 s, transferred to the GC using a pyrolysis transfer line (350°C) and injected onto the GC using a split ratio of 5:1; the injector port temperature was maintained at 350°C. The oven was programmed to heat at 4°C/min from 40°C (held for 4 min) to 300°C (held for 15 min). The mass spectrometric detector (MSD) mass range scanned from was *m/z* 60–600 at one scan per second and there was a solvent delay of 4 min. Compounds were identified using the National Institute of Standards and Technology (NIST) database and by comparison with spectra from the literature.

### FTIR

Samples of the fossil tissues and a natural melanin standard (*Sepia officinalis*) were compressed onto the surface of individual pellets of indium. IR spectra of the sample were collected using a Perkin Elmer Spectrum Two spectrometer (wavenumber range 500–4000 cm^−1^) in attenuated total reflectance mode with a resolution of 4 cm^−1^. Absorption bands were identified with reference to the NIST reference library and published literature.

### ToF‐SIMS

ToF‐SIMS analysis was performed on a BioToF‐SIMS instrument (Kore Technology Ltd, Ely, UK) using a 20 keV Au_3_^+^ primary ion beam (Ionoptika Ltd, Chandlers Ford, UK). The samples were mounted in indium for analysis. Samples of fossil soft tissues and associated sediment were cleaved immediately prior to analysis using a sterile scalpel. The sample of melanin standard was prepared as a powder crushed in indium. The 500 pA primary ion beam was pulsed (120 ns) and scanned over the sample and data acquired in negative ion mode. Secondary ions were extracted into the dual‐stage reflectron mass analyser using a delayed extraction field of 2500 V/cm, and detected with a post‐acceleration voltage of 7 kV. Charge compensation was provided by a 50 eV electron floodgun (100 nA, 100 μs pulses). The instrument was operated with a mass resolution *m*/Δ*m c*. 1000 and a lateral resolution *c*. 1 μm. Images comprising 256 × 256 pixels of total ion signal or selected *m*/*z* values were acquired from fossil frog samples over an area ranging from 200 × 200 μm^2^ to 1000 × 1000 μm^2^. All analyses were conducted under static SIMS conditions with a primary ion fluence <10^12^ ion cm^−2^ to ensure ion beam damage did not contribute to the measured signal. Optical images of the analysed areas were recorded to allow subsequent SEM imaging of the corresponding regions.

## Results

### Py‐GCMS

**Figure 2:**
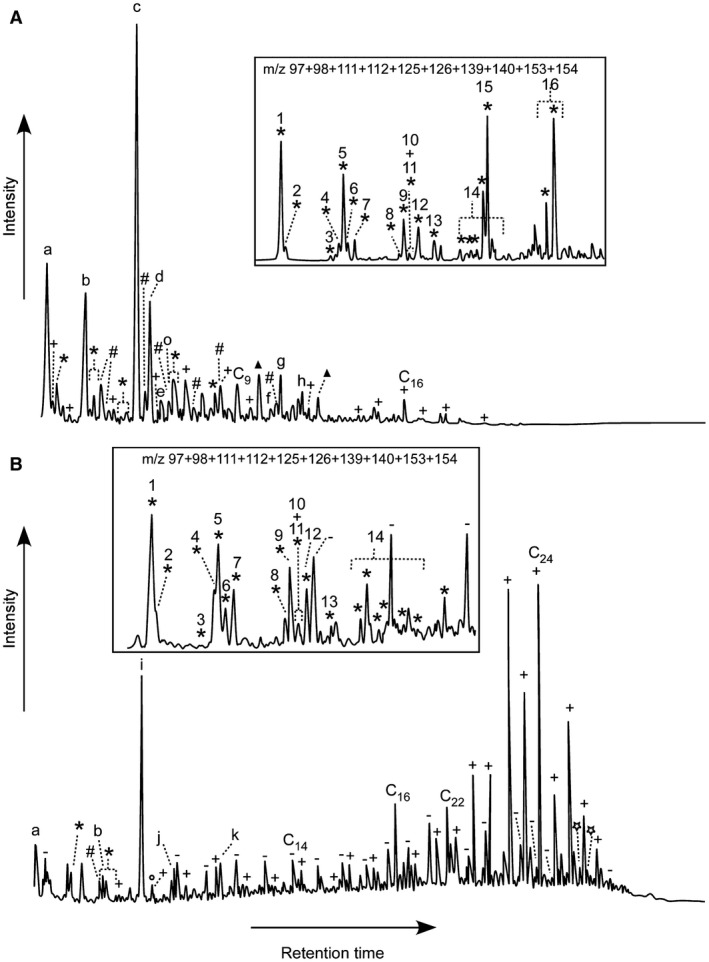
Partial TMAH‐assisted Py‐GCMS total ion current chromatograms of Libros microstructures (A) and matrix (B); insets (not to scale) show the *m/z* 97 + 98 + 111 + 112 + 125 + 126 + 139 + 140 + 153 + 154 chromatograms of dotted areas revealing the presence and distribution of alkylated thiophene moieties. *, alkylated thiophene; +, fatty acid methyl ester; –, *n*‐alkane/*n*‐alkene doublets; #, benzene derivative; ○, phenol derivative; ▲, benzoic acid derivative; 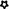, hopane moieties; a, dimethyl disulfide; b, methylsulfonic acid methyl ester; c, propenoic acid butyl ester; d, succinic acid dimethylester; e, methyl succinic acid dimethyl ester; f, methyl indolinone; g, phthalate; h, 2,5‐dimethoxy benzoic acid methyl ester (or isomer); i, contaminant; j, methyl phenylsulfide; k, 3,5‐dimethyl‐2‐(methylsulfanyl) thiophene. c_x_ indicates the carbon chain length and numbers refer to the alkylated thiophenes listed in Table 1.

The pyrogram of the fossil microbodies is dominated by propenoic acid butyl ester (compound ‘c’ in Fig 2A) and a range of organic sulfur compounds (OSCs), including dimethyl disulfide (compound ‘a’ in Fig. 2A), methylsulfonic acid methyl ester (compound ‘b’ in Fig. 2A) and a series of short chain alkylated thiophenes (Fig. 2A; Table 1). Also present are an extended series of fatty acid methyl esters that range up to C_19_ and have maxima at C_9_ and/or C_16_, and derivatives of benzene, phenol and benzoic acid. Notably, a series of *n*‐alkane‐/alkene doublets and bacterially‐derived biomarkers, such as hopanoid pyrolysis products, are absent. In contrast, analyses of the host sediment produced a pyrogram dominated by extended series of *n‐*alkane‐/alkene doublets and fatty acid methyl esters that range up to C_29_ and have a maximum at C_24_ (Fig. 2B; Table 1). Hopanes and OSCs, predominantly short chain alkylated thiophenes, are also present, but are relatively minor components.

**Table 1:** Alkylated thiophenes identified in the TMAH‐assisted pyrolysates of Libros microstructures and sedimentary matrix

1	2‐methylthiophene
2	3‐methylthiophene
3	2‐ethylthiophene
4	2,5‐dimethylthiophene
5	2,4‐dimethylthiophene
6	2,3‐dimethylthiophene
7	3,4‐dimethylthiophene
8	2‐propylthiophene
9	2‐ethyl‐5‐methylthiophene
10	2‐ethyl‐4‐methylthiophene
11	Ethylmethylthiophene
12	2,3,5‐trimethylthiophene
13	2,3,4‐trimethylthiophene
14	C_8_H_12_S†
15	C_6_H_6_O_2_S‡
16	C_7_H_8_O_2_S§

Numbers refer to Fig. 2; †Mixture of 2‐methyl‐5‐propylthiophene, 2,5‐diethylthiophene, 2‐butylthiophene, 2‐ethyl‐3,5‐dimethylthiophene, ethyldimethylthiophene and/or 5‐ethyl‐2,3‐dimethylthiophene; ‡Mixture of methyl‐2‐thiophene carboxylate and methyl‐3‐thiophene carboxylate; §Mixture of methyl‐5‐methyl‐2‐thiophene carboxylate and methyl‐3‐methyl‐2‐thiophene carboxylate.

### FTIR

**Figure 3:**
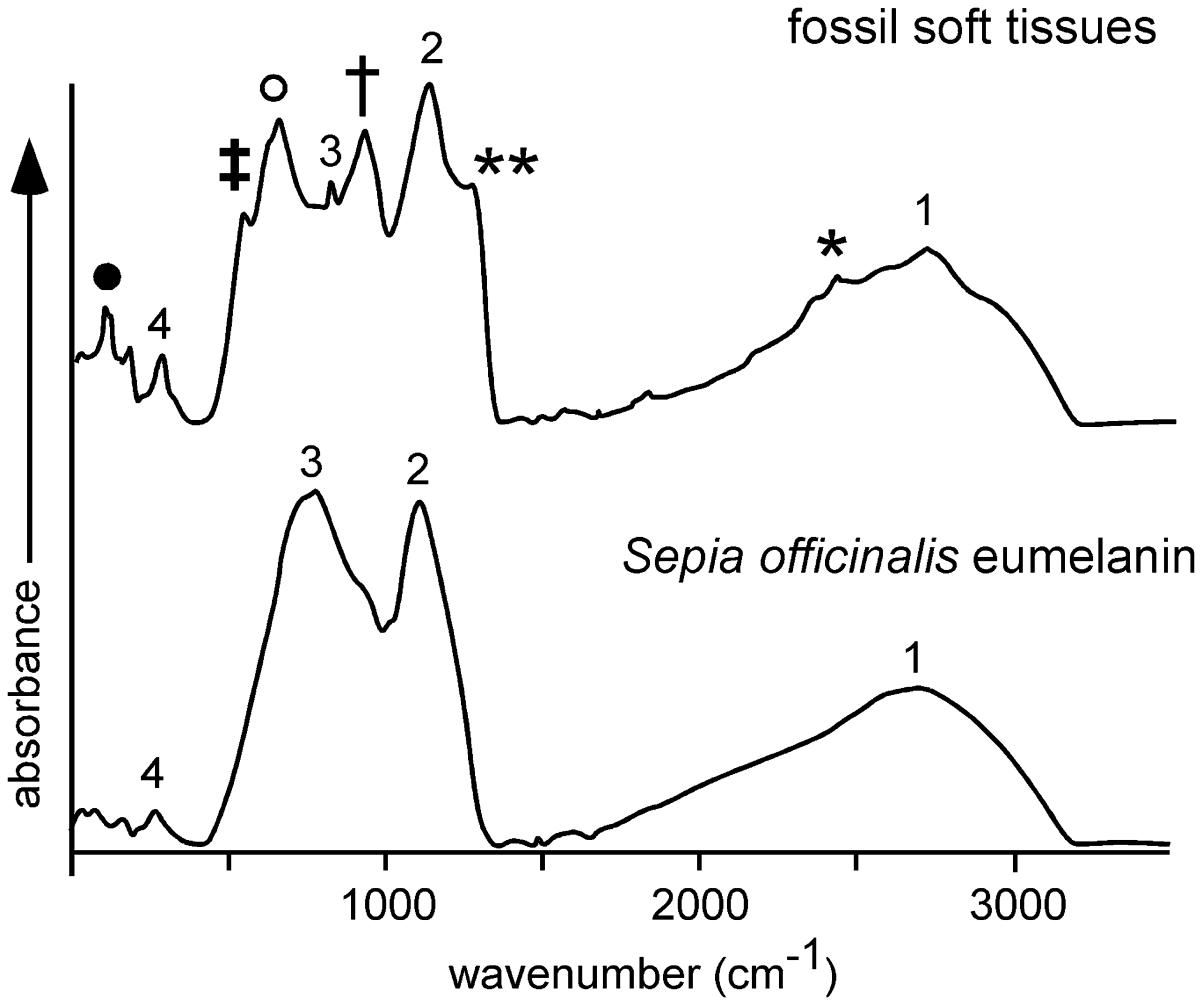
FTIR absorption spectra for the fossil microbodies in the Libros frogs and in a melanin standard (*Sepia officinalis*). Details of the absorption bands and associated symbols are in Table 2.

The measured FTIR spectra reveal a close similarity between the preserved organic functional groups present in the fossil microbodies and the melanin standard (Fig. 3, Table 2). The FTIR spectrum of the latter is dominated by three major absorption bands (Hong & Simon 2006; Centeno *et al*. 2008; Glass *et al*. 2014). The broad peak centred at 3200 cm^−1^ corresponds to absorption due to the stretching mode of the OH bond. The relatively sharp bands at 1606 cm^−1^ and 1290 cm^−1^ correspond to the carbonyl C=O stretching vibration, probably in indole quinone, and a combination of OH and NH in‐plane bending vibrations, respectively. The minor band at 775 cm^−1^ may reflect bending modes of aromatic C‐H or C=C bonds.

**Table 2:** Infrared peak assignments for the fossil microbodies and a melanin standard (*Sepia officinalis*)

Notation in Fig. 3	Bond	Wavenumber (cm^−1^)	Mode of vibration	Melanin standard	Fossil microbodies
1	O‐H	3200–3207	Stretch	X	X
2	C=O	1606–1620	Stretch	X	X
3	O‐H ± N‐H	1290–1315	Bend	X	X
4	C‐H (aromatic)	775–780	Bend	X	X
*	C‐H (aliphatic)	2920	Stretch	–	X
**	C=O (ketone)	1713	Stretch	–	X
†	C‐H (aliphatic)	1428	Bend	–	X
‡	C‐S ± C‐O	1040	Stretch	–	X
◦	C=S	1152	Stretch	–	X
●	C‐S	598	–	–	X

Numbers and symbols refer to Fig. 3.

These absorption bands also dominate FTIR spectra of the fossil microbodies (Fig. 3; Table 2). In addition, the fossil spectra also show a major band at 1152 cm^−1^ and minor bands at 2920 cm^−1^, 1713 cm^−1^, 1428 cm^−1^, 1040 cm^−1^ and 598 cm^−1^. Several of these bands can be attributed to the presence of sulfur: the major band at 1152 cm^−1^ corresponds to the C=S stretching vibration and the minor bands at 1040 cm^−1^ and 598 cm^−1^ may represent stretching vibrations in CS functional groups (Bloxham *et al*. 2002); CO stretching may also contribute to the band at 1040 cm^−1^. The minor band at 2920 cm^−1^ is due to stretching vibrations in CH_x_ functional groups, probably lipids (Glass *et al*. 2014); this is consistent with the minor band at 1428 cm^−1^, which reflects the bending mode of CH bonds in CH_2_ or CH_3_ groups. The band at 1713 cm^−1^ is attributed to the carbonyl ketone stretch and may indicate lower levels of indole quinone in the fossil sample than in the melanin standard (Glass *et al*. 2014).

### ToF‐SIMS

**Figure 4:**
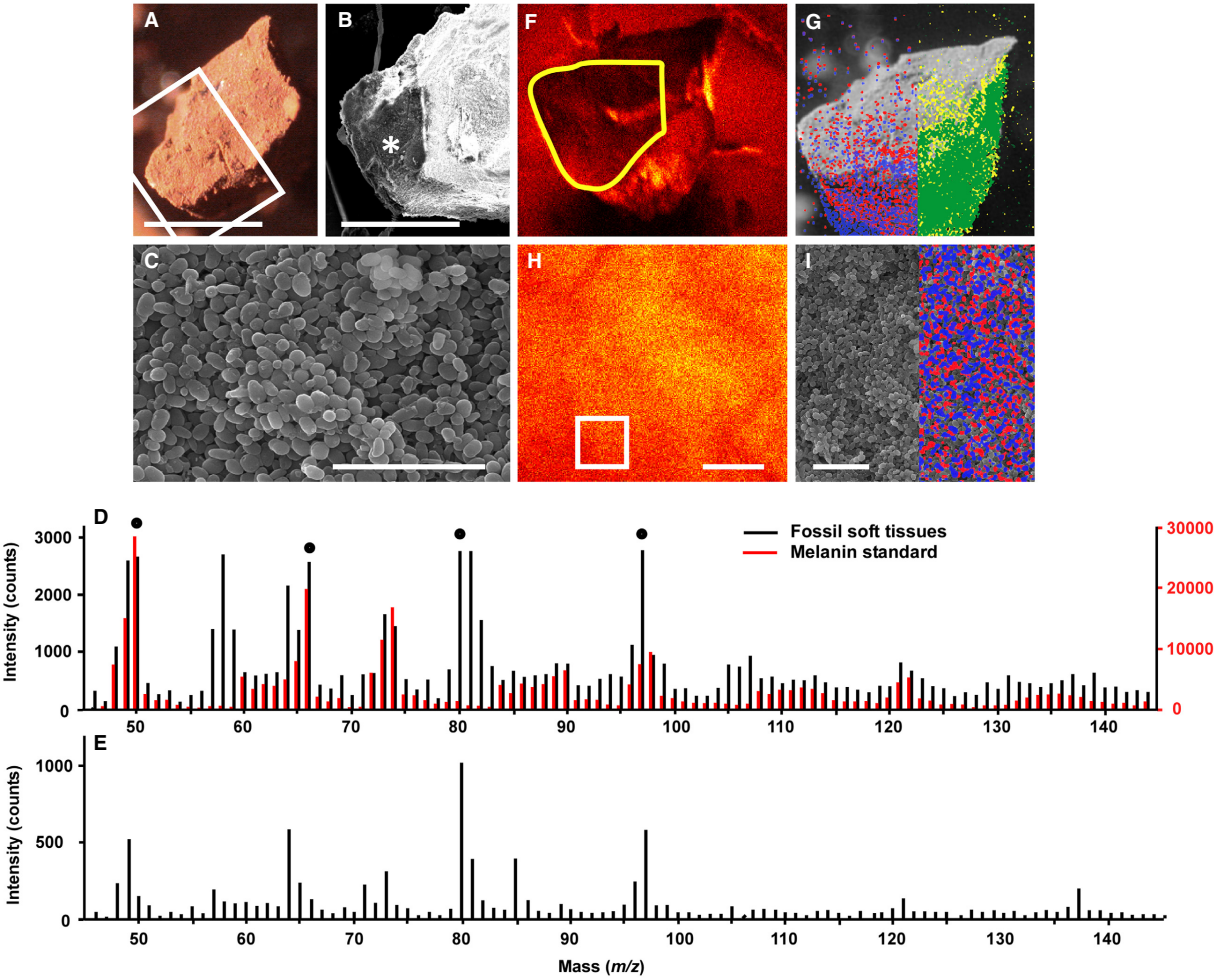
Melanin in Miocene frogs from Libros. A–C, light micrograph (A) and scanning electron micrographs (B–C) of freshly fractured sample of soft tissues from MNCN 63663. B, detail of area indicated by box in A. C, detail of area indicated by * in B, showing densely packed, spheroidal to ovoid microbodies. D, negative ion ToF‐SIMS spectra for the region of the fossil tissue sample highlighted in yellow in F, and for a synthetic melanin standard; filled circles indicate ions used for ToF‐SIMS images in G and I. E, negative ion ToF‐SIMS spectrum for a sample of sediment from the area indicated by * in Fig. 1A. F, H, ToF‐SIMS images of total ion counts; F, sample shown in A; H, a selected region of the counterpart of the sample. G, I, ion images showing the distribution of ions derived from melanin (blue: *m/z* 50; red: *m/z* 66) and organosulfur compounds (yellow: *m/z* 80; green: *m/z* 97) superimposed onto scanning electron micrographs; G, is the sample in A; I, shows a detailed view of densely packed microbodies from the area indicated by a box in H; the signal is strongest in the lower part of the sample as this region was orientated towards the detector. Scale bars represent: 500 μm (A); 300 μm (B); 4 μm (C); 5 μm (H–I).

The samples analysed using ToF‐SIMS comprise exclusively ovoid microstructures (Fig. 4A–C). There is a very close agreement between the spectra from the fossil microbodies and the melanin standard, in terms of both the position and relative intensity of peaks (Fig. 4D). Key peaks in the spectrum for the melanin standard at *m/z* 50, 66, 73, 74, 97, 98, 121 and 122 reflect the nitrogen‐rich chemical structure of melanin (Lindgren *et al*. 1998). These are also among the most prominent peaks in the fossil microbodies (Fig. 4D). Additional peaks at *m/z* 80 and *m/z* 97 in the fossil sample correspond to SO_3_^−^ and HSO_4_^−^ and also occur in the host sediment (Fig. 4E). Almost all other major peaks in the spectrum from the fossil soft tissues can be tentatively assigned to fragment ions consistent with the structure of nitrogen‐rich melanins (Table 3). Further, ion images showing the spatial signal intensity distribution for key melanin peaks (*m/z* 50 and 66) superimposed on SEM images demonstrate a clear correlation between the presence of the fossil microbodies and the peaks for melanin (Fig. 4F–I). With the exception of the peaks at *m/z* 80 and 97 (see above), ToF‐SIMS spectra from the sediment and fossil tissues are notably dissimilar; none of the key peaks in the melanin standard are prominent in the sediment (Fig. 4D–E). The dominant peaks in the sediment spectrum correspond to carbon chains of various length, some possibly bearing O or N; proteinaceous moieties have been previously identified in the organic matter from the Libros oil shales (del Río *et al*. 2003b).

**Table 3:** Tentative assignments and *m/z* ratios for peaks in negative ToF‐SIMS spectra of melanin and microstructures in the Libros frogs

Tentative assignment	Theoretical mass (u)	Melanin standard	Fossil frog	Sediment
C_4_	48.00	48.00	48.00	47.98
C_4_H	49.01	49.01	49.02	49.00
C_3_N	50.00	50.00	50.03	50.00
C_3_HN	51.01	51.01	51.03	51.02
C_3_H_2_N	52.02	52.02	52.02	52.01
C_5_	60.00	60.00	60.01	59.99
C_5_H	61.01	61.01	61.00	61.00
C_4_N/C_5_H_2_	62.00/62.02	62.01	62.02	62.00
C_4_HN	63.01	63.01	63.02	63.00
C_4_H_2_N/CH_4_O_3_	64.02/64.02	64.01	63.98	63.96
C_4_HO/H_3_NO_3_	65.00/65.01	65.01	65.02	65.00
C_3_NO	66.00	66.00	66.02	65.99
C_6_	72.00	72.00	72.00	72.00
C_6_H/C_2_H_3_NO_2_	73.01/73.02	73.01	73.02	73.01
C_5_N/C_2_H_2_O_3_	74.00/74.00	74.00	74.02	74.01
SO_3_	79.96	79.98	79.97	79.96
HSO_3_	80.97	80.99	80.98	80.98
C_7_	84.00	84.00	84.00	84.00
C_7_H	85.01	85.01	85.01	85.02
C_6_N/C_7_H_2_	86.00/86.02	86.01	86.01	86.00
C_6_HN	87.01	87.01	87.02	87.02
C_6_H_2_N/C_3_H_4_O_3_	88.02/88.02	88.02	88.02	87.99
C_6_HO/C_2_H_3_NO_3_	89.00/89.01	89.01	89.02	88.99
C_5_NO	90.00	90.00	90.01	90.00
C_7_H_7_	91.06	91.02	91.02	91.01
C_8_	96.00	96.02	95.96	95.97
HSO_4_/C_4_H_3_NO_2_	96.96/97.02	97.01	96.97	96.98
C_7_N/C_4_H_2_O_3_	98.00/98.00	98.00	98.00	98.00
C_9_	108.00	108.00	108.00	108.00
C_9_H	109.01	109.01	109.00	108.99
C_8_N/C_9_H_2_	110.00/110.02	110.01	110.01	110.01
C_8_HN	111.01	111.01	111.02	111.02
C_8_H_2_N/C_5_H_4_O_3_	112.02/112.02	112.02	112.00	111.98
C_8_HO/C_4_H_3_NO_3_	113.00/113.01	113.02	112.99	112.99
C_7_NO	114.00	114.02	114.01	114.00
C_10_	120.00	120.00	119.99	112.00
C_10_H/C_6_H_3_NO_2_	121.01/121.02	121.01	121.00	120.98
C_9_N	122.00	122.00	121.99	121.98
C_12_H/C_8_H_3_NO_2_	145.01/145.02	145.01	144.99	144.96

Grey shading highlights key N‐bearing molecular ions likely to be derived from melanin.

## Discussion

### The nature of the fossil microbodies

The microbodies associated with the soft tissues of the Libros frogs were originally interpreted as fossilized bacteria on the basis of their size, geometry, restriction to the soft tissues of the specimens, and the presence of conjoined microbodies suggestive of cell mitosis (McNamara *et al*. 2012). As others have noted (Zhang *et al*. 2010), the size and geometry of the microbodies from the fossil amphibians cannot be considered to be definitive. The structures range between *c*. 0.4 μm and 1.0 μm in width (Fig. 1B; McNamara *et al*. 2012); this falls within the range of structures identified as melanosomes in other fossil vertebrates (Li *et al*. 1984; Zhang *et al*. 2010; Lindgren *et al*. 1998) but alone does not exclude their being bacteria. The fossil microbodies envelop the bones and the stomach contents (McNamara *et al*. 2012), i.e. they occur on both ventral and dorsal surfaces of the body, and most are located internal to the layer of phosphatized skin. This distribution is consistent with their being melanosomes (Zhang *et al*. 2010), but not an external ‘microbial overgrowth’ *sensu* Moyer *et al*. (2010). Further, there is no evidence in the host sediments for benthic microbial mats (McNamara *et al*. 2009). The seemingly connected microbodies resembling bacterial cells in the process of fission could simply represent closely juxtaposed melanosomes. Indeed, the dense packing of microbodies in the Libros amphibians and of melanosomes reported from other fossil taxa (Clarke *et al*. 2008; Vinther *et al*. 2010; Zhang *et al*. 2010; Barden *et al*. 2011; Glass *et al*. 2014; Lindgren *et al*. 1998, 2011) is notable. This could originate via degradation and collapse of more labile soft tissues that enveloped the melanosomes *in vivo*, thus forming a layer of melanosomes that does not retain the original spatial arrangement and density of melanosomes in the tissue (and may even derive from different tissues; M.E. McNamara, J.S. Kaye, M.J. Benton, P.J. Orr & N.P. Lockyer, unpub. data). Notably in the case of the adult frogs from Libros, the microbodies external to the phosphatized skin show a markedly different distribution on the dorsal and ventral surfaces. Microbodies are typically densely packed on the dorsal surface of the specimen, but sparse on the ventral surface. This distribution is consistent with the distribution of integumentary melanosomes in the integument of many extant frogs, which generates countershading (Beebee & Griffiths 2000).

In addition to these data on anatomical and sedimentary context, the microbodies from the Libros amphibians exhibit key ultrastructural features that support their being melanosomes. In unstained and stained TEM sections, the fossil microbodies are uniformly electron‐dense (Fig. 1E); electron density is enhanced, but remains uniform among microbodies, in stained sections (Fig. 1F). In stained TEM sections, melanosomes in extant vertebrates are also uniformly electron‐dense (Prum 2013a; Hellström *et al*. 2006; Moyer *et al*. 2010), but bacteria exhibit a marginal, electron‐dense cell wall surrounding a relatively electron‐lucent cell lumen (Mesnage *et al*. 2009; Saar‐Dover *et al*. 1998). A similar ultrastructure (i.e. an electron‐dense margin surrounding an electron‐lucent core) is present in some of the microbodies from the Libros amphibians (Fig. 1G). This feature is only evident, however, in microbodies located immediately adjacent to (i.e. within *c*. 5 μm of) the phosphatized skin. The electron‐dense margin of these microbodies has been interpreted as a diagenetic precipitate of calcium phosphate, with the phosphate ions derived from the decaying skin (which is rich in calcium phosphate *in vivo*; McNamara *et al*. 2012). These microbodies exhibit a nanocrystalline texture on their surfaces (Fig. 1H) (McNamara *et al*. 2012) and are rich in Ca and P, in contrast to the other, uniformly electron‐dense, microbodies, which are carbonaceous in composition (McNamara *et al*. 2012). Many of the microbodies from the Libros amphibians exhibit electron‐lucent inclusions in TEM images (Fig. 1E) that are strikingly similar to the internal vacuoles of melanosomes in extant vertebrate tissues (Akazaki *et al*. 2014; Moyer *et al*. 2010), but are rare in bacteria. Further, the carbonaceous fossil microbodies from Libros typically exhibit subtle granular to botryoidal surface nanotextures (Fig. 1C) that resemble those of modern melanosomes (Moyer *et al*. 2010).

Our new chemical data provide strong evidence that the preserved microbodies in the Libros frogs are not fossil bacteria. Neither Py‐GCMS nor ToF‐SIMS analyses reveal evidence of bacterially derived compounds. This is not a taphonomic artefact: bacterially‐derived hopane moieties are present in the host sediment. Hopanoid producing microbes are predominantly aerobic; abundant hopane moieties in other fossil vertebrate soft tissues has been interpreted as evidence of extended decay in aerobic conditions (Edwards *et al*. 2010). The lack of hopane moieties in the Libros frog tissues could indicate predominantly anaerobic decay before and after deposition. The presence of hopanes in the host sediment could reflect slower rates of settling of organic particles through the water column and thus more prolonged decay in aerobic waters. Further, FTIR and ToF‐SIMS analyses reveal a strong spectral agreement between the fossil tissues and the melanin standard (Figs 3, 4D); ToF‐SIMS data also demonstrate that key peaks for melanin are localized to the fossil microbodies (Fig. 4F–I). These data confirm that the microbodies contain high concentrations of melanin and/or its derivatives. Both Py‐GCMS and ToF‐SIMS analyses reveal striking differences between the chemical composition of the fossil microbodies in the Libros frogs and that of the sedimentary matrix. In particular, the microbodies and sediment differ markedly in the abundance of *n‐*alkane‐/alkene doublets and in the fatty acid methyl ester distribution patterns and maxima evident in the pyrograms, and in peak position and intensity in the ToF‐SIMS spectra. These data strongly indicate that the melanin‐based, and other, organic compounds in the microbodies are indigenous and are not derived from the host sediment. Collectively, the context, ultrastructure and chemical composition of the fossil microbodies in the Libros amphibians are strong evidence that the fossil microbodies in the Libros frogs are fossil melanosomes.

### Preservation through sulfurization (natural vulcanization)

Py‐GCMS and ToF‐SIMS analyses confirm the presence of diverse OSCs in the Libros melanosomes and the sedimentary matrix; this is supported by FTIR spectra, which indicate the presence of various sulfur moieties not present in the eumelanin standard. These OSCs are unlikely to reflect the presence of S‐bearing phaeomelanins as typical pyrolysis products of phaeomelanin, for example, S‐bearing compounds such as benzothiazine and benzothiazole (Lindgren *et al*. 2011) were not detected. Further, ToF‐SIMS spectra for phaeomelanin show prominent peaks for S‐bearing molecular fragments at *m/z* 57, 58, 81, 82, 105, 106, 118 and 134 (Lindgren *et al*. 2011). With the exception of the peak at *m/z* 81 (which we have attributed to HSO_3_), none of these peaks are prominent in our fossil samples. Small amounts of sulfur can occur within natural eumelanins, but their origins are unclear (Jimbow *et al*. 2006; Prota 1992) and they are unlikely to be responsible for the high abundance and diversity of OSCs in the Libros melanosomes. Many of the OSCs in the Libros melanosomes are also present in the host sediment, although in lower abundance, where a melanin origin is highly improbable. Thus the OSCs in the Libros melanosomes most likely indicate the presence of sulfurized organic matter. This confirms previous suggestions (McNamara *et al*. 2012) that the carbonaceous soft tissues in the Libros amphibians are preserved via sulfurization. The presence of abundant sulfurized organic matter in the sedimentary matrix is consistent with previous reports of OSCs in the sedimentary kerogen (del Río *et al*. 2003b) and confirms that sulfurization of organic matter was widespread in the Libros sediments. The extended series of fatty acid methyl esters also present in the fossil soft tissues and sedimentary matrix indicates that part of the organic matter is preserved via polymerization processes that did not involve the incorporation of sulfur.

S‐mediated reduction processes are critical to the preservation of organic matter in sediments (Hebting *et al*. 2006). Sulfurization of organic matter is characteristic of environments where inputs of bacterially generated sulfide exceed the availability of reactive iron (Sinninghe Damsté *et al*. 1992). Low concentrations of reactive iron are, however, not required for sulfurization: certain organic molecules are predisposed to sulfurization and can be sulfurized very early during diagenesis, even within the water column (Sinninghe Damsté *et al*. 1992; Melendez *et al*. 2006, *b*). Sulfurization may therefore occur under a broad spectrum of depositional conditions; unsurprisingly, OSCs have been reported from various lacustrine and marine settings from the Permian to the Recent (Sinninghe Damsté *et al*. 2012, 1992, 1997, 2004; Grice *et al*. 1992, 2004; van Kaam‐Peters *et al*. 2000; Kok *et al*. 2003, *b*; Werne *et al*. 1984; Kolonic *et al*. 2013; van Dongen *et al*. 1999; Jaraula *et al*. 2009). Sulfurization of organic molecules in the sedimentary kerogen and fossil melanosomes from Libros is attributed to deposition beneath anoxic, sulfidic bottom waters poor in reactive iron (del Río *et al*. 2003b; McNamara *et al*. 2012). Such conditions are likely to characterize other fossil deposits in which melanosomes are preserved. Indeed, the presence of elevated levels of sulfur and, especially, of OSCs in fossil melanosomes associated with other vertebrate and invertebrate fossils (see above) strongly suggests preservation via sulfurization. These fossils are hosted within diverse sediments, including freshwater lacustrine varves (Xiagou Formation, China), volcanic maar oil shales (Oligocene of Enspel, Germany), marine diatomite (Fur Formation, Denmark) and marine oil shales (Cretaceous Boquillas Formation; Jurassic of Dorset, UK). Geochemical analyses of the host sediments at these and other localities offer a test for the possibility that sulfurization occurred and thus constrain the confidence with which potential phaeomelanosomes can be identified in fossils.

Melanin is a large and relatively inert biopolymer (Riley 2013b). Other biopolymers, e.g. cellulose, lignin and chitin, are not usually the primary contributors to the preservation of organic soft tissues in fossils as the process of *in situ* polymerization involves primarily endogenous lipids (Gupta *et al*. 2011; Gupta & Briggs 1998). Sulfurization, however, is known to affect biomolecules of different recalcitrance, including carbohydrates, lipids, and structural biopolymers, including melanin (Jimbow *et al*. 2006; Melendez *et al*. 2006, *b*). In pyrograms of the Libros melanosomes, the high abundance and distribution pattern of short‐chain alkylated (C_0_‐C_5_) thiophenes resemble those of sulfurized monosaccharides (carbohydrates; Sinninghe Damsté *et al*. 2006; Kok *et al*. 2006; van Dongen *et al*. 1998a) and kerogen from the Kimmeridge Clay Formation (van Kaam‐Peters & Sinninghe Damsté 2007, van Kaam‐Peters *et al*. 2000; van Dongen *et al*. 1999). Diagenetic sulfurization is known to generate a spectrum of organic compounds (i.e. low molecular weight OSCs and macromolecular S‐bearing aggregates) that are resistant to bacterial degradation and remineralization and are thus preserved in the fossil record (Valisolalao *et al*. 1993; Brassell *et al*. 1986; Sinninghe Damsté & de Leeuw 2003; Sinninghe Damsté *et al*. 1992, *b*). Preservation via sulfurization is therefore likely to enhance the inherent resistance (Goldstein *et al*. 2013) of melanins to microbial degradation.

## Conclusions

Although the anatomical and sedimentological context of the microbodies associated with the soft tissues of the Libros frogs is not conclusive, their geochemical composition (dominated by melanin and distinct from the host sediment) confirms that the fossil microbodies represent fossilized melanosomes. The high abundance of OSCs in the fossil melanosomes confirms that the latter are preserved via diagenetic incorporation of sulfur, i.e. preservation through sulfurization. This process is likely to enhance the inherent recalcitrance and thus preservation potential of melanin. Given that sulfurization can occur in various marine and freshwater depositional settings, and that many examples of fossil melanosomes are associated with sulfur and/or organosulfur compounds, this mode of preservation is likely to contribute widely to preservation of melanosomes in the fossil record. Future studies of sulfur‐rich fossil melanosomes will require the host sediment to be tested for geochemical evidence of sulfurization in order to constrain interpretations of potential phaeomelanosomes and thus of original integumentary colour in fossils.

## References

[pala12238-bib-0001] Akazaki, S. , Takahashi, T. , Nakano, Y. , Nidshida, T. , Mori, H. , Takaoka, A. , Aoki, H. , Chen, H. , Kunisada, T. and Koike, K. 2014 Three‐dimensional analysis of melanosomes isolated from B16 melanoma cells by using ultra high voltage electron microscopy. Microscopy Research, 2, 42286, 8.

[pala12238-bib-0002] Anadón, P. , Rosell, L. and Talbot, M. R. 1992 Carbonate replacement of lacustrine gypsum deposits in two Neogene continental basins. Sedimentary Geology, 78, 201–216.

[pala12238-bib-0003] Barden, H. E. , Wogelius, R. A. , Li, D. , Manning, P. L. , Edwards, N. P. and van Dongen, B. E. 2011 Morphological and geochemical evidence of eumelanin preservation in the feathers of the Early Cretaceous bird, *Gansus yumenensis* . PLoS One, 6, e25494. 22022404 10.1371/journal.pone.0025494 PMC3192724

[pala12238-bib-0004] Barden, H. E. , Bergmann, U. , Edwards, N. P. , Egerton, V. M. , Manning, P. L. , Perry, S. , van Veelen, A. , Wogelius, R. and van Dongen, B. E. 2014 Bacteria or melanosomes? A geochemical analysis of microbodies on a tadpole from the Oligocene Enspel Formation of Germany. Paleodiversity & Paleoenvironments, 95, 33–45.

[pala12238-bib-0005] Beebee, T. and Griffiths, R. 2000 Amphibians and reptiles: a natural history of the British herpetofauna. Harper Collins, London, 270 pp.

[pala12238-bib-0006] Bloxham, S. , Eicher‐Lorka, O. , Jakubenas, R. and Niaura, G. 2002 Surface‐enhanced Raman spectroscopy of ethanethiol adsorbed at copper electrode. Chemija, 13, 185–189.

[pala12238-bib-0007] Brassell, S. C. , Lewis, C. A. , de Leeuw, J. W. , De Lange, F. and Sinninghe Damsté, J. S. 1986 Isoprenoid thiophenes: novel products of sediment diagenesis? Nature, 320, 160–162.

[pala12238-bib-0008] Briggs, D. E. G. 1999 Molecular taphonomy of animal and plant cuticles: selective preservation and diagenesis. Philosophical Transactions of the Royal Society of London B, 354, 7–16.

[pala12238-bib-0009] Carney, R. M. , Vinther, J. , Shawkey, M. D. , D'Alba, L. and Ackermann, J. 2012 New evidence on the colour and nature of the isolated *Archaeopteryx* feather. Nature Communications, 3, 637–643. 10.1038/ncomms1642 22273675

[pala12238-bib-0010] Centeno, S. A. and Shamir, J. 2008 Surface enhanced Raman scattering (SERS) and FTIR characterization of the sepia melanin pigment used in works of art. Journal of Molecular Structure, 873, 149–159.

[pala12238-bib-0011] Clarke, J. A. , Ksepka, D. T. , Salas‐Gismondi, R. , Altamirano, A. J. , Shawkey, M. D. , D'Alba, L. , Vinther, J. , Devries, T. J. and Baby, P. 2010 Fossil evidence for evolution of the shape and color of penguin feathers. Science, 330, 954–957. 20929737 10.1126/science.1193604

[pala12238-bib-0012] Colleary, C. , Dolocan, A. , Gardner, J. , Singh, S. , Wuttke, M. , Rabenstein, R. , Habersetzer, J. , Schaal, S. , Feseha, M. , Clemens, M. , Jacobs, B. F. , Currano, E. D. , Jacobs, L. L. , Sylvestersen, R. L. , Gabbott, S. E. and Vinther, J. V. 2015 Chemical, experimental, and morphologial evidence for diagenetically altered melanin in exceptionally preserved fossils. Proceedings of the National Academy of Sciences, 112, 12592–12597. 10.1073/pnas.1509831112 PMC4611652 26417094

[pala12238-bib-0013] van Dongen, B. E. , Schouten, S. and Sinninghe Damsté, J. S. 2003a Sulfurization of carbohydrates results in a S‐rich, unresolved complex mixture in kerogen pyrolysates. Energy & Fuels, 17, 1109–1118.

[pala12238-bib-0014] van Dongen, B. E. , Schouten, S. , Baas, M. , Geenevasen, J. A. J. and Sinninghe Damsté, J. S. 2003b An experimental study of the low‐temperature sulfurization of carbohydrates. Organic Geochemistry, 34, 1129–1144.

[pala12238-bib-0015] van Dongen, B. E. , Schouten, S. and Sinninghe Damsté, J. S. 2006 Preservation of carbohydrates through sulfurization in a Jurassic euxinic shelf sea: examination of the Blackstone Band TOC cycle in the Kimmeridge Clay Formation, UK. Organic Geochemistry, 37, 1052–1073.

[pala12238-bib-0016] Dutta, S. , Hartkopf‐Fröder, C. , Mann, U. , Wilkes, H. , Brocke, R. and Bertram, N. 2010 Macromolecular composition of Palaeozoic scolecodonts: insights into the molecular taphonomy of zoomorphs. Lethaia, 43, 334–343.

[pala12238-bib-0017] Edwards, N. P. , Barden, H. E. , van Dongen, B. E. , Manning, P. L. , Larson, P. L. , Bergmann, U. , Sellers, W. I. and Wogelius, R. A. 2011 Infrared mapping resolves soft tissue preservation in 50 million year‐old reptile skin. Proceedings of the Royal Society B, 278, 3209–3218. 21429928 10.1098/rspb.2011.0135 PMC3169023

[pala12238-bib-0018] Edwards, N. P. , Manning, P. L. , Bergmann, U. , Larson, P. L. , van Dongen, B. E. , Sellers, W. I. , Webb, S. M. , Sokaras, D. , Alonso‐Mori, R. , Ignatyev, K. , Barden, H. E. , van Veelen, A. , Anné, J. , Egerton, V. M. and Wogelius, R. A. 2014 Leaf metallome preserved over 50 million years. Metallomics, 6, 774–782. 24804302 10.1039/c3mt00242j

[pala12238-bib-0019] Glass, K. , Ito, S. , Wilby, P. R. , Sotad, T. , Nakamurad, A. , Bowers, C. R. , Vinther, J. , Dutta, S. , Summons, R. , Briggs, D. E. G. , Wakamatsu, K. and Simon, J. D. 2012 Direct chemical evidence for eumelanin pigment from the Jurassic period. Proceedings of the National Academy of Sciences, 109, 10218–10223. 10.1073/pnas.1118448109 PMC3387130 22615359

[pala12238-bib-0020] Glass, K. , Ito, S. , Wilby, P. R. , Sota, T. , Nakamura, A. , Bowers, C. R. , Miller, K. E. , Dutta, S. , Summons, R. E. , Briggs, D. E. G. , Wakamatsu, K. and Simon, J. D. 2013 Impact of diagenesis and maturation on the survival of eumelanin in the fossil record. Organic Geochemistry, 64, 29–37.

[pala12238-bib-0021] Goldstein, G. , Flory, K. R. , Browne, B. A. , Majid, S. , Ichida, J. M. and Burtt, E. H. Jr 2004 Bacterial degradation of black and white feathers. Auk, 121, 656–659.

[pala12238-bib-0022] de Graaf, W. , Sinninghe Damsté, J. S. and de Leeuw, J. W. 1992 Laboratory simulation of natural suplhurization: I. Formation of monomeric and oligomeric isoprenoud polysulphides by low‐temperature reactions of inorganic polysulphides with phytol and phytadienes. Geochimica et Cosmochimica Acta, 56, 4321–4328.

[pala12238-bib-0023] Grice, K. , Schouten, S. , Nissenbaum, A. , Charach, J. and Sinninghe‐Damsté, J. S. 1998 A remarkable paradox: freshwater algal (*Botryococcus braunii*) lipids in an ancient hypersaline euxinic ecosystem. Organic Geochemistry, 28, 195–216.

[pala12238-bib-0024] Grice, K. , Schaeffer, P. , Schwark, L. and Maxwell, J. R. 1996 Molecular indicators of palaeoenvironmental conditions in an immature Permian shale (Kupferschiefer, Lower Rhine Basin, N.W. Germany) from free and sulfide‐bound lipids. Organic Geochemistry, 25, 131–147.

[pala12238-bib-0025] Gupta, N. S. and Briggs, D. E. G. 2011 Taphonomy of animal organic skeletons through time 199–221. *In* Allison P. A. and Bottjer D. J. (eds). Taphonomy: process and bias through time. Springer, Dordrecht, 612 pp.

[pala12238-bib-0026] Gupta, N. S. , Michels, R. , Briggs, D. E. G. , Evershed, R. P. and Pancost, R. D. 2006 The organic preservation of fossil arthropods: an experimental study. Proceedings of the Royal Society of London B, 273, 2777–2783. 10.1098/rspb.2006.3646 PMC1635499 17015325

[pala12238-bib-0027] Gupta, N. S. , Cody, G. D. , Tetlie, O. E. , Briggs, D. E. G. and Summons, R. E. 2009 Rapid incorporation of lipids into macromolecules during experimental decay of invertebrates: initiation of geopolymer formation. Organic Geochemistry, 40, 589–594.

[pala12238-bib-0028] Hebting, Y. , Schaeffer, P. , Behrens, A. , Adam, P. , Schmit, G. , Schneckenburger, P. , Bernasconi, S. M. and Albrecht, P. 2006 Biomarker evidence for a major preservation pathway of sedimentary organic carbon. Science, 312, 1627–1631. 16690819 10.1126/science.1126372

[pala12238-bib-0029] Hellström, A. , Watt, B. , Fard, S. S. , Tenza, D. , Manström, P. , Narfström, K. , Ekesten, B. , Ito, S. , Wakamatsu, K. , Larsson, J. , Ulfendahl, M. , Kullander, K. , Raposo, G. , Kerje, S. , Halböök, F. , Marks, M. S. and Andersson, L. 2011 Inactivation of Pmel alters melanosome shape but has only a subtle effect on visible pigmentation. PLoS Genetics, 7, e1002285. 21949658 10.1371/journal.pgen.1002285 PMC3174228

[pala12238-bib-0030] de las Heras, F. X. C. , Anadón, P. and Cabrera, L. 2003 Biomarker record variations in lacustrine coals and oil shales: contribution from Tertiary basins in NE Spain 187–228. *In* Valero Garcés B. L. (ed.). Limnogeology in Spain: a tribute to Kerry Kelts. Biblioteca de Ciencias: Spanish Research Council (Consejo Superior de Investigaciones Científicas (CSIC)), Madrid, 439 pp.

[pala12238-bib-0031] Hong, L. and Simon, J. D. 2006 Insight into the binding of divalent cations to sepia eumelanin from IR absorption spectroscopy. Photochemistry & Photobiology, 82 (5), 1265–1269. 16696594 10.1562/2006-02-23-RA-809

[pala12238-bib-0032] Jaraula, C. M. B. , Grice, K. , Twitchett, R. J. , Böttcher, M. E. , le Metayer, P. , Dastidar, A. G. and Opazo, L. F. 2013 Elevated pCO_2_ leading to Late Triassic extinction, persistent photic zone euxinia, and rising sea levels. Geology, 41, 955–958.

[pala12238-bib-0033] Jimbow, K. , Miyake, Y. , Homma, K. , Yasuda, K. , Izumi, Y. , Tsutsumi, A. and Ito, S. 1984 Characterization of melanogenesis and morphogenesis of melanosomes by physicochemical properties of melanin and melanosomes in malignant melanoma. Cancer Research, 44, 1128–1134. 6318981

[pala12238-bib-0034] van Kaam‐Peters, H. M. E. and Sinninghe Damsté, J. S. 1997 Characterisation of an extremely organic sulphur‐rich, 150 Ma old carbonaceous rock: palaeoenvironmental implications. Organic Geochemistry, 27, 371–397.

[pala12238-bib-0035] van Kaam‐Peters, H. M. E. , Schouten, S. , Köster, J. and Sinninghe Damsté, J. S. 1998 Controls on the molecular and carbon isotopic composition of organic matter deposited in a Kimmeridgian euxenic shelf sea: evidence for preservation of carbohydrates through sulfurisation. Geochimica et Cosmochimica Acta, 62, 3259–3283.

[pala12238-bib-0036] Knight, T. K. , Bingham, S. , Lewis, R. D. and Savrda, C. E. 2011 Feathers of the Ingersoll Shale, Eutaw Formation (Upper Cretaceous), Eastern Alabama: the largest collection of feathers from North American Mesozoic Rocks. Palaios, 26, 364–376.

[pala12238-bib-0037] Kok, M. D. , Rijpstra, W. I. C. , Robertson, L. , Volkman, J. K. and Sinninghe Damsté, J. S. 2000a Early steroid sulfurization in surface sediments of a permanently stratified lake (Ace Lake, Antarctica). Geochimica et Cosmochimica Acta, 64, 1425–1436.

[pala12238-bib-0038] Kok, M. D. , Schouten, S. and Sinninghe Damsté, J. S. 2000b Formation of insoluble, nonhydrolyzable, sulfur‐rich macromolecules via incorporation of inorganic sulfur species into algal carbohydrates. Geochimica et Cosmochimica Acta, 64, 2689–2699.

[pala12238-bib-0039] Kolonic, S. , Sinninghe Damsté, J. S. , Böttcher, M. E. , Kuypers, M. M. M. , Kuhnt, W. , Beckmann, B. , Scheeder, G. and Wagner, T. 2002 Geochemical characterization of Cenomanian/Turonian black shales from the Tarfaya Basin (SW Morocco): relationships between palaeoenvironmental conditions and early sulphurization of sedimentary organic matter. Journal of Petroleum Geology, 25, 325–350.

[pala12238-bib-0040] Li, Q. , Gao, K.‐Q. , Vinther, J. , Shawkey, M. D. , Clarke, J. A. , D'Alba, L. , Meng, Q. , Briggs, D. E. G. and Prum, R. O. 2010 Plumage color patterns of an extinct dinosaur. Science, 327, 1369–1372. 20133521 10.1126/science.1186290

[pala12238-bib-0041] Li, Q. , Gao, K.‐Q. , Meng, Q. , Clarke, J. A. , Shawkey, M. D. , D'Alba, L. , Pei, R. , Ellison, M. , Norell, M. A. and Vinther, J. 2012 Reconstruction of *Microraptor* and the evolution of iridescent plumage. Science, 335, 1215–1219. 22403389 10.1126/science.1213780

[pala12238-bib-0042] Lindgren, J. , Uvdal, P. , Sjövall, P. , Nilsson, D. E. , Engdahl, A. , Schultz, B. P. and Thiel, V. 2012 Molecular preservation of the pigment melanin in fossil melanosomes. Nature Communications, 3, 824–831. 10.1038/ncomms1819 22569368

[pala12238-bib-0043] Lindgren, J. , Sjovall, P. , Carney, R. M. , Uvdal, P. , Gren, J. A. , Dyke, G. , Schultz, B. P. , Shawkey, M. D. , Barnes, K. R. and Polcyn, M. J. 2014 Skin pigmentation provides evidence of convergent melanism in extinct marine reptiles. Nature, 506, 484–486. 24402224 10.1038/nature12899

[pala12238-bib-0044] Manning, P. L. , Morris, P. M. , Mcmahon, A. , Jones, E. , Gize, A. , Macquaker, J. H. S. , Wolff, G. , Thompson, A. , Marshall, J. , Taylor, K. G. , Lyson, X. X. , Gaskall, S. , Reamtong, O. , Sellers, W. I. , van Dongen, B. E. , Buckley, M. and Wogelius, R. A. 2009 Mineralized soft‐tissue structure and chemistry in a mummified hadrosaur from the Hell Creek Formation, North Dakota (USA). Proceedings of the Royal Society B, 276, 3429–3437. 19570788 10.1098/rspb.2009.0812 PMC2817188

[pala12238-bib-0045] Manning, P. L. , Edwards, N. P. , Wogelius, R. A. , Bergmann, U. , Barden, H. E. , Larson, P. L. , Schwarz Wings, D. , Egerton, V. M. , Sokaras, D. , Mori, R. A. and Sellers, W. I. 2013 Synchrotron‐based chemical imaging reveals plumage patterns in a 150 million year old early bird. Journal of Analytical Atomic Spectrometry, 28, 1024–1030.

[pala12238-bib-0046] Manning, P. L. , Wogelius, R. A. , van Dongen, B. E. , Lyson, T. R. , Bergmann, U. , Webb, S. , Buckley, M. , Egerton, V. M. and Sellers, W. I. 2014 The role and biochemistry of melanin pigment in the exceptional preservation of hadrosaur skin 600–610. *In* Eberth D. A. and Evans D. C. (eds). Hadrosaurs. Indiana University Press, 619 pp.

[pala12238-bib-0047] McGraw, K. J. 2006 Mechanics of melanin‐based coloration 243–294. *In* Hill G. and McGraw K. (eds). Bird coloration. Vol. 1: mechanisms and measurements. Harvard University Press, Cambridge, MA, 589 pp.

[pala12238-bib-0048] McNamara, M. E. , Orr, P. J. , Kearns, S. L. , Alcalá, L. , Anadón, P. and Peñalver, E. 2006 High‐fidelity organic preservation of bone marrow in c. 10 million year old amphibians. Geology, 34, 641–644.

[pala12238-bib-0049] McNamara, M. E. , Orr, P. J. , Kearns, S. L. , Alcalá, L. , Anadón, P. and Peñalver, E. 2009 Soft tissue preservation in Miocene frogs from Libros (Spain): insights into the genesis of decay microenvironments. Palaios, 24, 104–117.

[pala12238-bib-0050] McNamara, M. E. , Orr, P. J. , Kearns, S. L. , Alcalá, L. , Anadón, P. and Peñalver, E. 2010 Organic preservation of fossil musculature with ultracellular detail. Proceedings of the Royal Society B, 277, 423–427. 19828545 10.1098/rspb.2009.1378 PMC2842642

[pala12238-bib-0051] McNamara, M. E. , Orr, P. J. , Kearns, S. L. , Alcalá, L. , Anadón, P. and Peñalver, E. 2012 What controls the taphonomy of exceptionally preserved taxa – environment or biology? A case study using exceptionally preserved frogs from the Miocene Libros Konservat‐Lagerstätte, Spain. Palaios, 27, 63–77.

[pala12238-bib-0052] McNamara, M. E. , Briggs, D. E. G. , Orr, P. J. , Field, D. and Wang, Z. 2013 Experimental maturation of feathers: implications for reconstructions of fossil feather colour. Biology Letters 9, 20130184. 23536445 10.1098/rsbl.2013.0184 PMC3645052

[pala12238-bib-0054] Melendez, I. , Grice, K. and Schwark, L. 2013a Exceptional preservation of Palaeozoic steroids in a diagenetic continuum. Scientific Reports, 3, 2768. 24067597 10.1038/srep02768 PMC3783881

[pala12238-bib-0055] Melendez, I. , Grice, K. , Trinajstic, K. , Ladjavardi, M. , Thompson, K. and Greenwood, P. F. 2013b Biomarkers reveal the role of photic zone euxinia in exceptional fossil preservation: an organic geochemical perspective. Geology, 41, 123–126.

[pala12238-bib-0056] Mesnage, S. , Tosi‐Couture, E. , Gounon, P. , Mock, M. and Fouet, A. 1998 The capsule and S‐Layer: two independent and yet compatible macromolecular structures in *Bacillus anthracis* . Journal of Bacteriology, 180, 52–58. 9422592 10.1128/jb.180.1.52-58.1998 PMC106848

[pala12238-bib-0057] Moyer, A. , Zheng, W. , Johnson, E. A. , Lamanna, M. C. , Li, D. , Lacovara, K. J. and Schweitzer, M. H. 2014 Alternative hypothesis for the origin of microbodies in fossil feathers. Scientific Reports, 4, 42331–42339. 10.1038/srep04233 PMC3942740 24595214

[pala12238-bib-0058] Ortí, F. , Rosell, L. and Anadón, P. 2003 Deep to shallow lacustrine evaporites in the Libros gypsum (southern Teruel Basin, Miocene, NE Spain): an occurrence of pelletal gypsum rhythmites. Sedimentology, 50, 361–386.

[pala12238-bib-0059] Pinheiro, F. L. , Horn, B. L. D. , Schultz, C. L. , de Andrade, J. A. F. G. and Sucerquia, P. A. 2012 Fossilized bacteria in a Cretaceous pterosaur headcrest. Lethaia, 45, 495–499.

[pala12238-bib-0060] Prota, G. 1992 Melanins and melanogenesis. Academic Press, New York.

[pala12238-bib-0061] Prum, R. 2006 Anatomy, physics, and evolution of structural coloration 295–353. *In* Hill G. and Mcgraw K. (eds). Bird coloration: function and evolution. Vol. II. Harvard University Press, 496 pp.

[pala12238-bib-0062] Riley, P. A. 1997 Melanin. International Journal of Biochemistry & Cell Biology, 29, 1235–1239. 9451820 10.1016/s1357-2725(97)00013-7

[pala12238-bib-0063] del Río, J. C. , Olivella, M. A. , Knicker, H. and de las Heras, F. X. C. 2004 Preservation of peptide moieties in three Spanish sulfur‐rich Tertiary kerogens. Organic Geochemistry, 35, 993–999.

[pala12238-bib-0064] Saar‐Dover, R. , Bitler, A. , Nezer, R. , Shmuel‐Galia, L. , Firon, A. , Shimoni, E. , Trieu‐Cuot, P. and Shai, Y. 2012 D‐alanylation of lipoteichoic acids confers resistance to cationic peptides in group B *Streptococcus* by increasing the cell wall density. PLoS Pathogens, 8, e1002891. 22969424 10.1371/journal.ppat.1002891 PMC3435245

[pala12238-bib-0065] Schouten, S. , de Graaf, W. , Sinninghe Damsté, J. S. , Van Driel, G. B. and de Leeuw, J. W. 1994 Laboratory simulation of natural sulphurization: II. Reaction of multifunctionalized lipids with inorganic polysulphides at low temperatures. Organic Geochemistry, 22, 825–834.

[pala12238-bib-0066] Sinninghe Damsté, J. S. and de Leeuw, J. W. 1990 Analysis, structure and geochemical significance of organically‐bound sulphur in the geosphere: state of the art and future research 1077–1101. *In* Durand B. and Behar F. (eds). Advances in organic geochemistry, 1989. Pergamon Press, Oxford.

[pala12238-bib-0067] Sinninghe Damsté, J. S. , de las Heras, F. X. C. , van Bergen, P. F. and de Leeuw, J. W. 1993 Characterization of Tertiary Catalan lacustrine oil shales: discovery of extremely organic sulphur rich Type I kerogens. Geochimica et Cosmochimica Acta, 57, 389–415.

[pala12238-bib-0068] Sinninghe Damsté, J. S. , Kohnen, M. E. L. and Horsfield, B. 1998a Origin of low‐molecular‐weight alkylthiophenes in pyrolysates of sulphur‐rich kerogens as revealed by micro‐scale sealed vessel pyrolysis. Organic Geochemistry, 29, 1891–1998.

[pala12238-bib-0069] Sinninghe Damsté, J. S. , Kok, M. D. , Köster, J. and Schouten, S. 1998b Sulfurized carbohydrates: an important sedimentary sink for organic carbon? Earth & Planetary Science Letters, 164, 7–13.

[pala12238-bib-0070] Sinninghe Damsté, J. S. , White, C. M. , Green, J. B. and de Leeuw, J. W. 1999 Organosulfur compounds in sulfur‐rich Rasa coal. Energy & Fuels, 13, 728–738.

[pala12238-bib-0071] Sinninghe Damsté, J. S. , Rijpstra, W. I. C. , Coolen, M. J. L. , Schouten, S. and Volkman, J. K. 2007 Rapid sulfurization of highly branched isoprenoid (HBI) alkenes in sulfidic Holocene sediments from Ellis Fjord, Antarctica. Organic Geochemistry, 38, 128–139.

[pala12238-bib-0072] Stankiewicz, B. A. , Briggs, D. E. G. , Michels, R. , Collinson, M. E. , Flannery, M. B. and Evershed, R. P. 2000 Alternative origin of aliphatic polymer in kerogen. Geology, 28, 559–562.

[pala12238-bib-0073] Tegelaar, E. W. , de Leeuw, J. W. , Derenne, S. and Largeau, C. 1989 A reappraisal of kerogen formation. Geochimica et Cosmochimica Acta, 53, 3103–3106.

[pala12238-bib-0075] Valisolalao, J. , Perakis, N. , Chappe, B. and Albrecht, P. 1984 A novel sulfur containing C35 hopanoid in sediments. Tetrahedron Letters, 25, 1183–1163.

[pala12238-bib-0076] Vinther, J. , Briggs, D. E. G. , Prum, R. O. and Saranathan, V. 2008 The colour of fossil feathers. Biology Letters, 4, 522–525. 18611841 10.1098/rsbl.2008.0302 PMC2610093

[pala12238-bib-0077] Vinther, J. , Briggs, D. E. G. , Clarke, J. , Mayr, G. and Prum, R. O. 2010 Structural coloration in a fossil feather. Biology Letters, 6, 128–131. 19710052 10.1098/rsbl.2009.0524 PMC2817243

[pala12238-bib-0078] Werne, J. P. , Hollander, D. J. , Behrens, A. , Schaeffer, P. , Albrecht, P. and Sinninghe Damsté, J. S. 2000 Timing of early diagenetic sulfurization of organic matter: a precursor‐product relationship in Holocene sediments of the anoxic Cariaco Basin, Venezuela. Geochimica et Cosmochimica Acta, 64, 1741–1751.

[pala12238-bib-0079] Wogelius, R. A. , Manning, P. L. , Barden, H. E. , Edwards, N. P. , Webb, S. M. , Sellers, W. I. , Taylor, K. G. , Larson, P. L. , Dodson, P. , You, H. , Da‐King, L. and Bergman, U. 2011 Trace metals as biomarkers for eumelanin pigment in the fossil record. Science, 333, 1622–1626. 21719643 10.1126/science.1205748

[pala12238-bib-0080] Zhang, F. , Kearns, S. L. , Orr, P. J. , Benton, M. J. , Zhou, Z. , Johnson, D. , Xu, X. and Wang, X. 2010 Fossilized melanosomes and the colour of Cretaceous dinosaurs and birds. Nature, 463, 1075–1078. 20107440 10.1038/nature08740

